# Supplementary material for: Anthropogenic forcing and response yield observed positive trend in Earth’s energy imbalance
Source: Nat Commun. 2021 Jul 28;12:4577. doi: 10.1038/s41467-021-24544-4 (PMC8319337; doi:10.1038/s41467-021-24544-4)
Supplement: Supplementary file 1 — Supplementary Information [file 41467_2021_24544_MOESM1_ESM.pdf]

Supplementary Information

Anthropogenic forcing and response yield observed positive trend in Earth's energy imbalance

Shiv Priyam Raghuraman[1*], David Paynter[2], V. Ramaswamy[2]

[1] Program in Atmospheric and Oceanic Sciences, Princeton University, Princeton, NJ, USA.
[2] NOAA Geophysical Fluid Dynamics Laboratory, Princeton, NJ, USA.
[*]email: shivr@princeton.edu

Supplementary Figures:

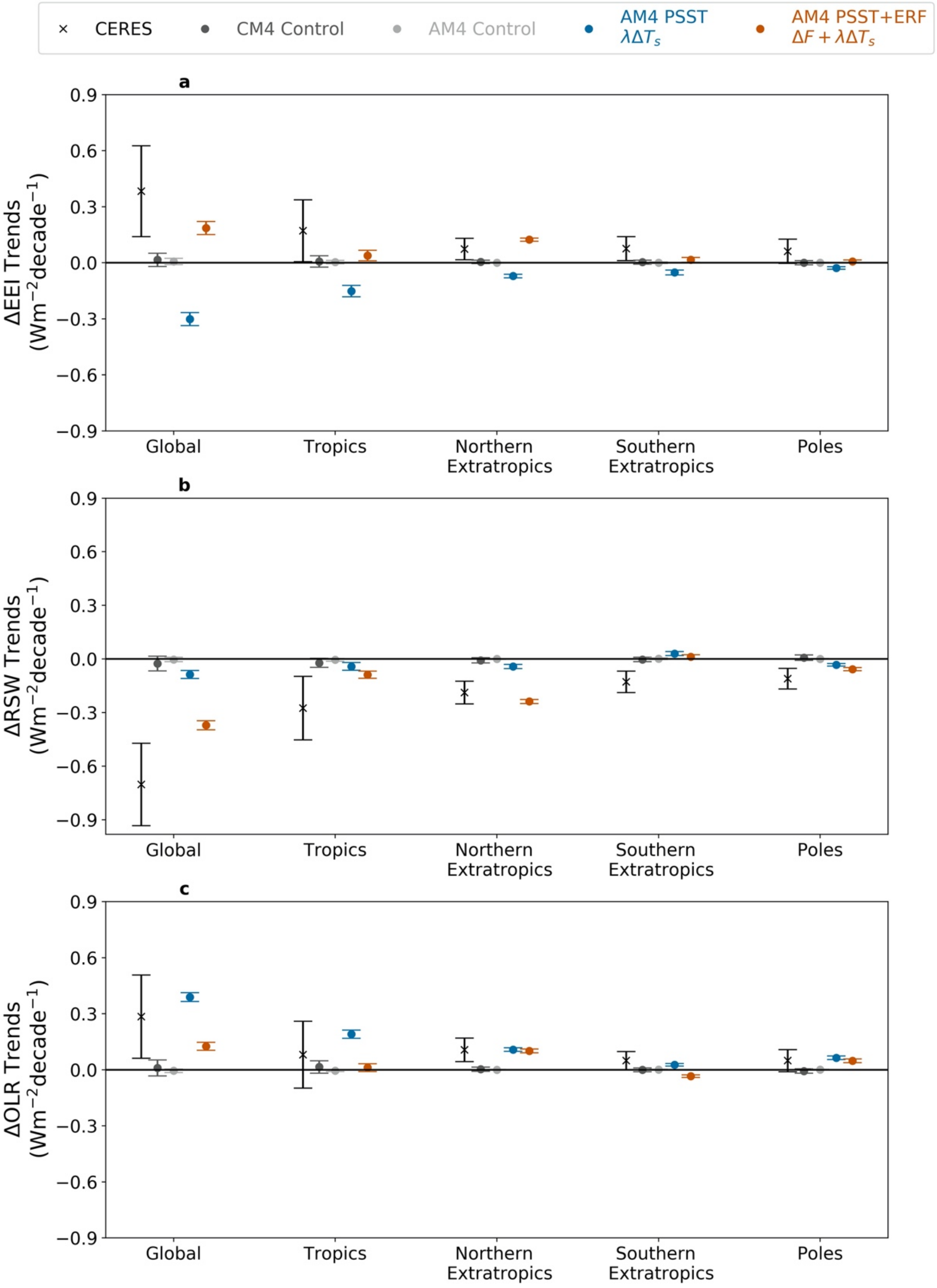

Supplementary Figure 1| Equivalent of Figure 2 but with 95% confidence intervals as estimates of uncertainty. For CERES, this is $1.96 \times \sqrt{\sigma_{Obs.}^{2} + \sigma_{Var.}^{2}}$ where $\sigma_{Var.}$ is the standard error associated with the linear fit of the time series multiplied. For the model ensembles, the 95% CI is $1.96 \times \sigma/\sqrt{n} \sim \epsilon/\sqrt{n}$ where n is the number of realizations per ensemble and σ is the standard

deviation of the realizations. Therefore, the model ensembles in this figure are identical to Figure 2 in main text but scaled by $\frac{1}{\sqrt{n}}$.

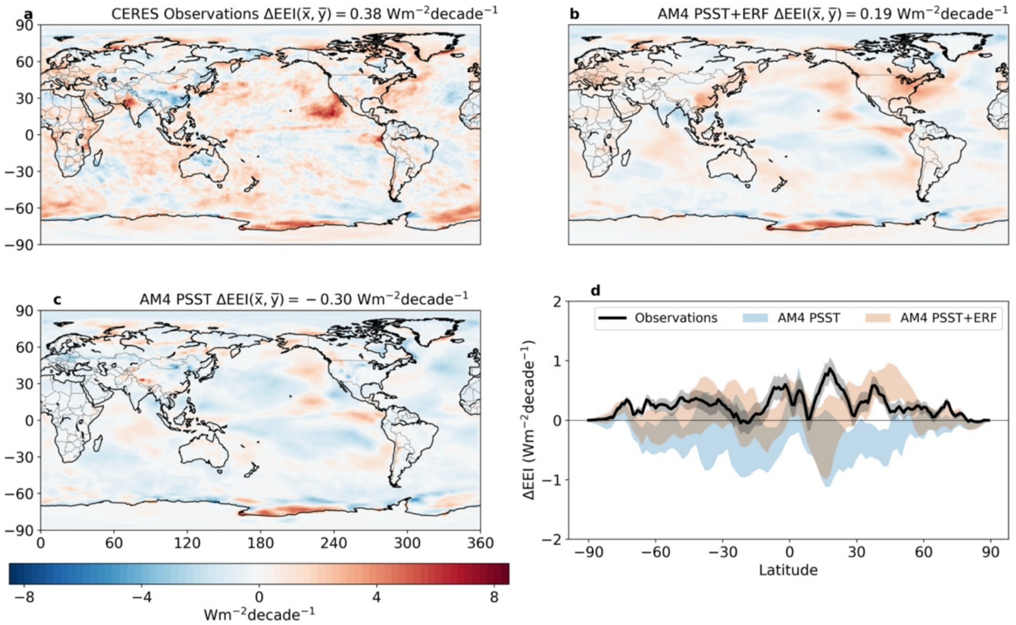

Supplementary Figure 2| Spatial and zonal trends in EEI. Model ensemble mean plotted in b and c. Model shading given by range of all realizations and trends are area weighted in d. CERES shading in d given by observational uncertainty. Positive indicates more energy in the system. In d, CERES = Clouds and the Earth's Radiant Energy System satellite observations (black), AM4 PSST = Prescribed sea surface temperatures (SSTs) and sea ice with forcing agents held fixed at 2014 levels in GFDL AM4 model (blue), and AM4 PSST+ERF = same as AM4 PSST but with forcing agents varying (orange). EEI = Earth's Energy Imbalance. Trend computed for anomalies time series (Δ).

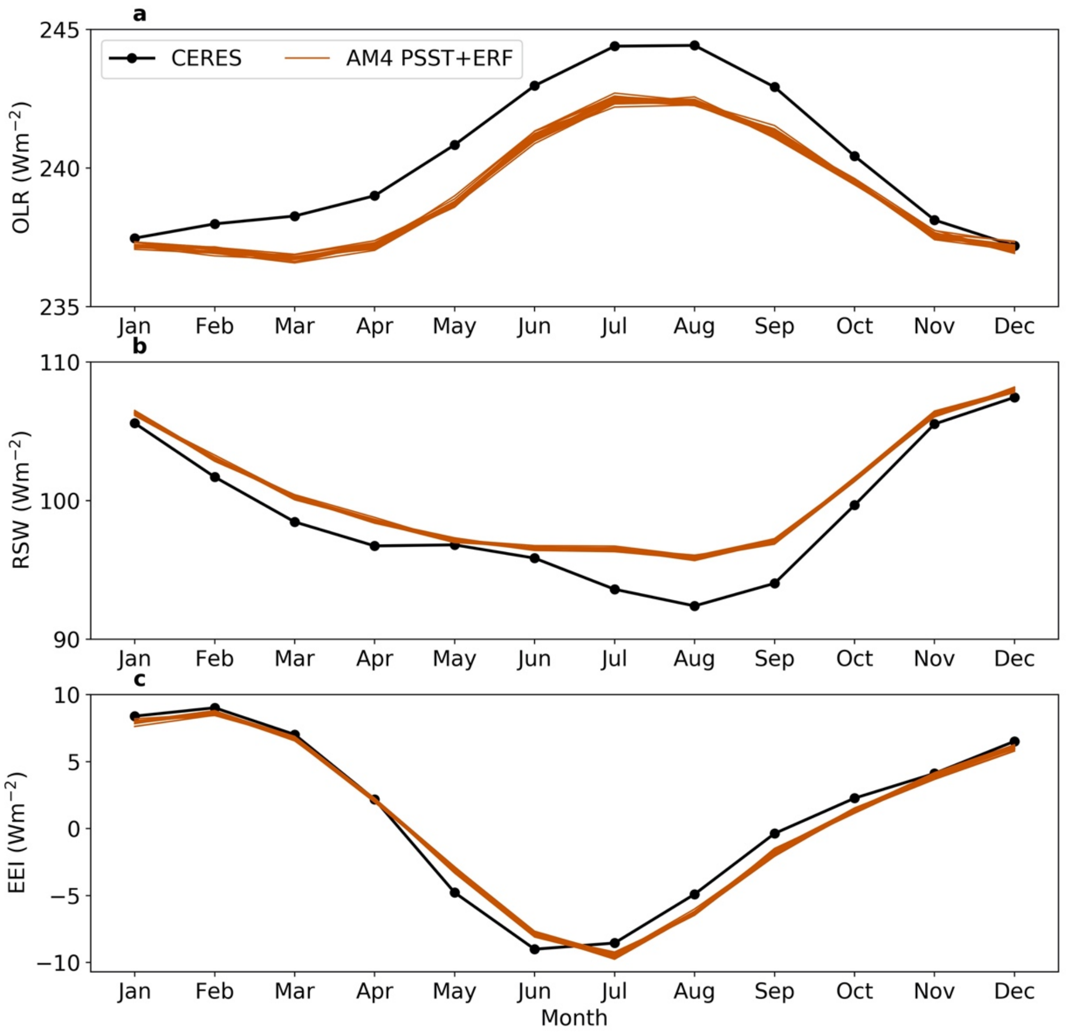

Supplementary Figure 3| Seasonal cycle of Earth's radiation budget. Validation of GFDL AM4 PSST+ERF experiment (all realizations plotted) against CERES observations. GFDL AM4 PSST+ERF = Geophysical Fluid Dynamics Laboratory Atmosphere Model 4.0 with Prescribed SSTs and sea ice with forcing agents varying. EEI = Earth's Energy Imbalance, RSW = Reflected Shortwave Radiation, and OLR = Outgoing Longwave Radiation.

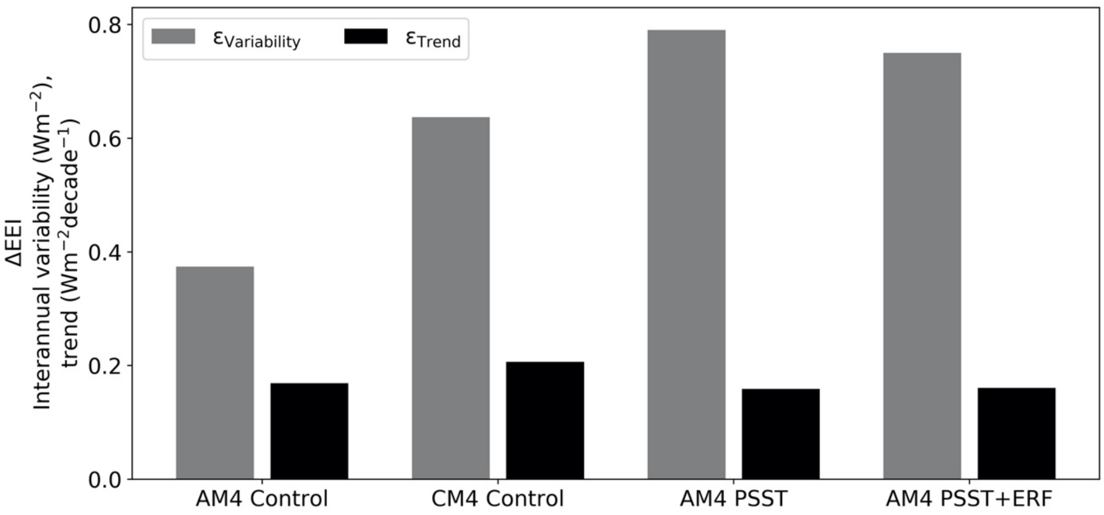

Supplementary Figure 4| Impact of SST variability on global EEI anomalies and trends. The $\epsilon_{\mathrm{Trend}}$ is the ϵ in Figure 2a and Equation (1), i.e., the 2σ spread of trends in EEI due to internal variability. We calculate $\epsilon_{\mathrm{Variability}}$ by computing the 2σ spread of anomalies (Δ) in EEI, i.e., our best estimate of interannual fluctuations. For the model experiments, since each realization has a value of $\epsilon_{\mathrm{Variability}}$, we plot the ensemble mean. For example, the mean of the 100 values of $\epsilon_{\mathrm{Variability}}$ of AM4 Control is 0.37 Wm^{-2}. This means that on average, 95% of the 20 years of ΔEEI falls within 0.37 Wm^{-2} of 0 (since mean of ΔEEI = 0). Note the increase in $\epsilon_{\mathrm{Variability}}$ as SST variability increases while $\epsilon_{\mathrm{Trend}}$ stays flat. AM4 PSST = Prescribed sea surface temperatures (SSTs) and sea ice with forcing agents held fixed at 2014 levels in GFDL AM4 model (blue), and AM4 PSST+ERF = same as AM4 PSST but with forcing agents varying (orange). CM4 = GFDL Coupled Model 4. EEI = Earth's Energy Imbalance.

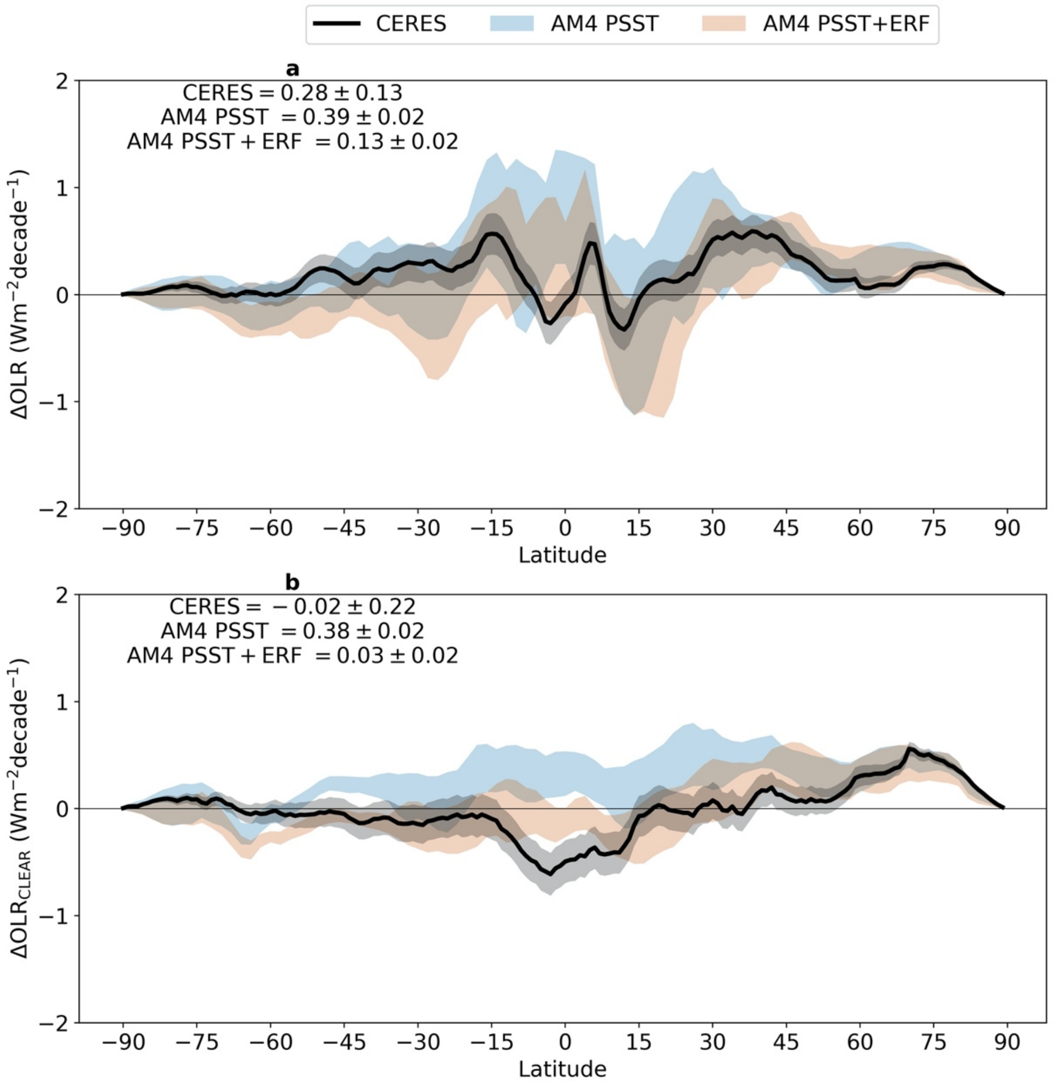

Supplementary Figure 5| Zonal mean trends in OLR, 2001-2020. a, Outgoing longwave radiation. **b,** Clear sky outgoing longwave radiation. Shading given by range of all realizations and trends are area weighted. CERES shading given by observational uncertainty. Negative values indicate more energy in the system while positive values indicate less energy in the system. Inset numbers listed are global mean values with 95% CI uncertainty, in units of $Wm^{-2}decade^{-1}$. Note that taking the difference between **a** and **b** yields the longwave cloud radiative effect. CERES = Clouds and the Earth's Radiant Energy System satellite observations (black), AM4 PSST = Prescribed sea surface temperatures (SSTs) and sea ice with forcing agents held fixed at 2014 levels in GFDL AM4 model (blue), and AM4 PSST+ERF = same as AM4 PSST but with forcing agents varying (orange). EEI = Earth's Energy Imbalance. Trend computed for anomalies time series (Δ).

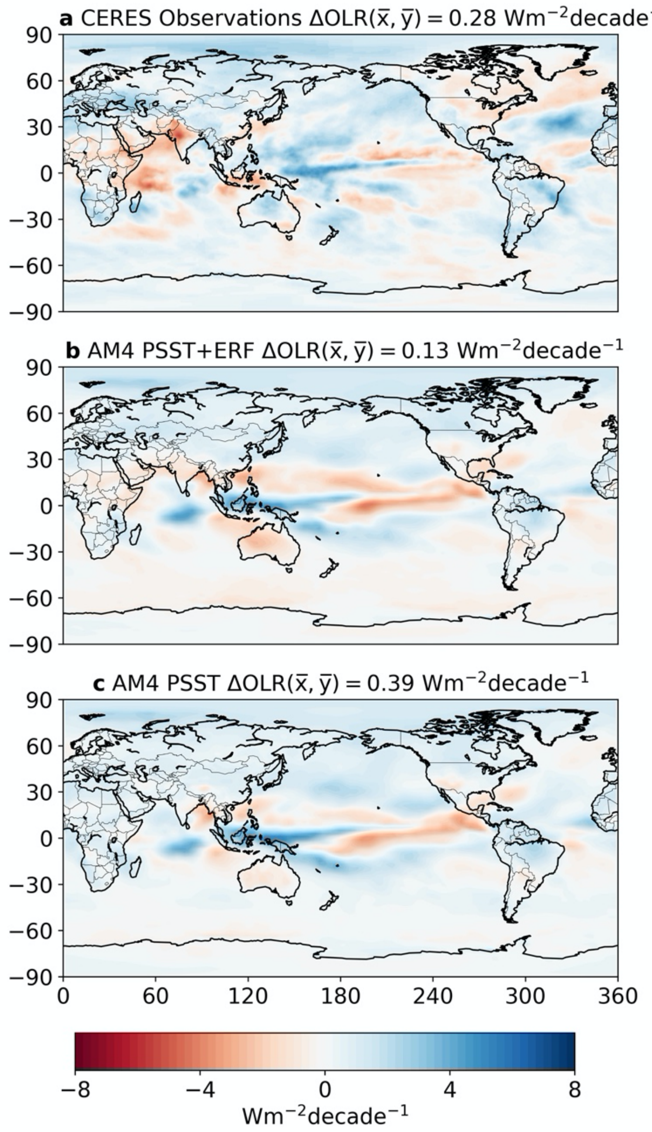

Supplementary Figure 6| Spatial map trends in OLR, 2001-2020. a, CERES. **b,** AM4 PSST+ERF ensemble mean. **c,** AM4 PSST ensemble mean. Positive indicates less energy in the system. CERES = Clouds and the Earth's Radiant Energy System satellite observations (black), AM4 PSST = Prescribed sea surface temperatures (SSTs) and sea ice with forcing agents held fixed at 2014 levels in GFDL AM4 model, and AM4 PSST+ERF = same as AM4 PSST but with forcing agents varying. OLR = Outgoing Longwave Radiation. Trend computed for anomalies time series (Δ).

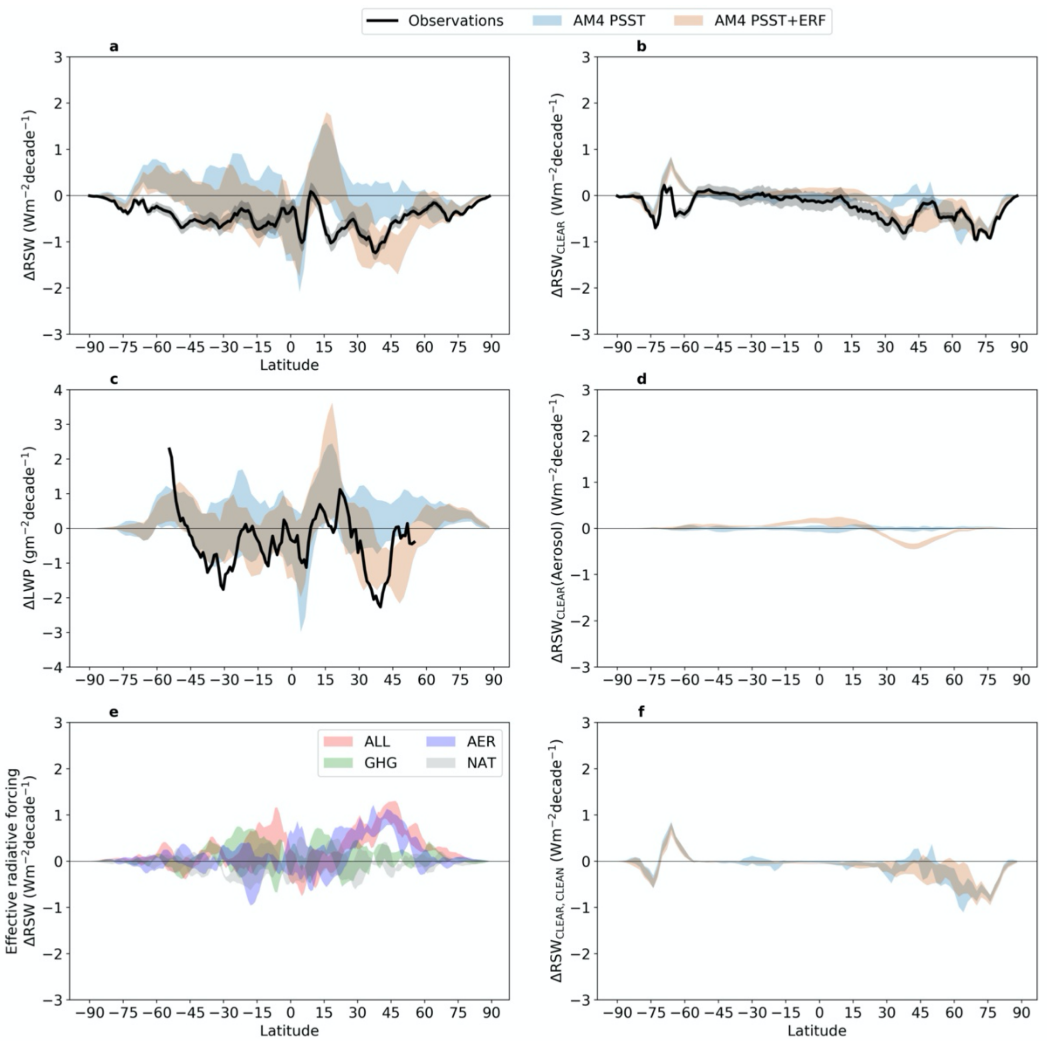

Supplementary Figure 7| Zonal mean trends, January 2001-November 2020. a, Reflected shortwave radiation. **b**, Clear sky reflected shortwave radiation. **c,** Liquid water path. **d**, Clear sky reflected shortwave radiation due to aerosol alone ($RSW_{CLEAR} - RSW_{CLEAR,CLEAN}$). **e**, Effective radiative forcing in reflected shortwave radiation in AM4 (ALL = all forcings, GHG = greenhouse gases only, AER = aerosol only, NAT = natural forcings only). **f**, Clear and clean sky reflected shortwave radiation. In **a-b,** observational data is derived from CERES (shading given by observational uncertainty), which calculates clear sky radiances for the entire grid box, consistent with models. Model shading given by range of all realizations and trends are area weighted. In **c,** observations are obtained from Moderate Resolution Imaging Spectroradiometer satellite data (MODIS). In **a, b, d, f,** negative values indicate more energy in the system while positive values indicate less energy in the system. In **e,** positive values indicate more energy in the system. CERES EBAF Ed4.1 "t" clear-sky and CRE fluxes, which are comparable with climate model outputs of these fluxes, is available till November 2020; this is why December 2020 is not included. CERES = Clouds and the Earth's Radiant Energy System satellite observations (black), AM4 PSST = Prescribed sea surface temperatures (SSTs) and sea ice with forcing agents held fixed at 2014 levels in GFDL AM4 model (blue), and AM4 PSST+ERF = same as AM4 PSST but with forcing agents varying (orange). Trend computed for anomalies time series (Δ).

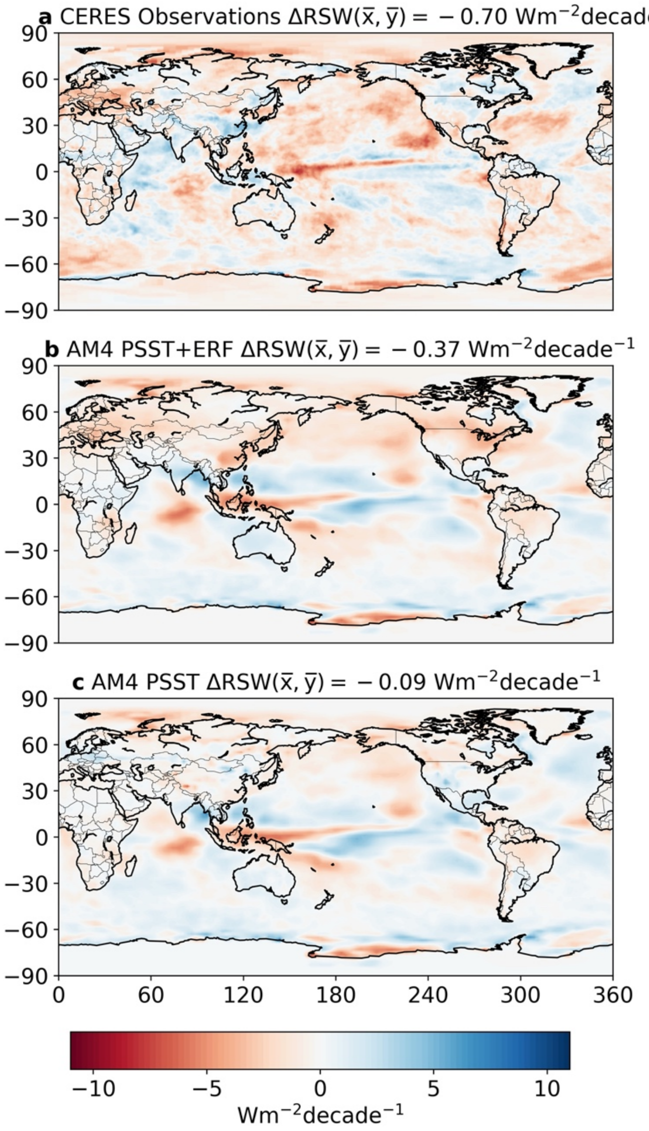

Supplementary Figure 8| Same as Supplementary Figure 6 but for RSW.

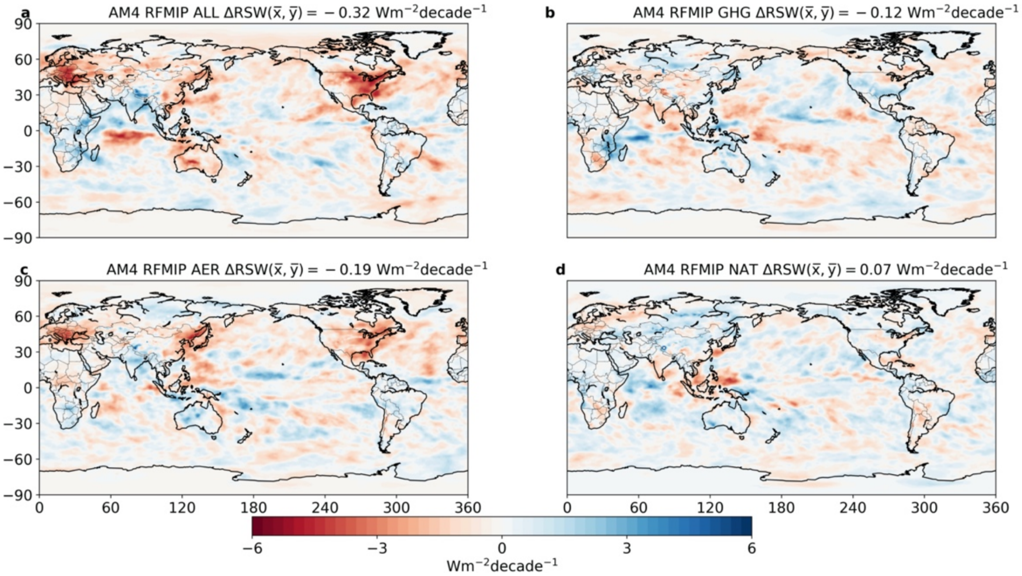

Supplementary Figure 9| ΔRSW trends in GFDL AM4 RFMIP ΔERF. **a,** ALL (all forcing agents). **b,** GHG (greenhouse gases only). **c,** AER (aerosols only). **d,** NAT (natural forcing agents only). Positive indicates less energy in the system. Ensemble means plotted. RFMIP = Radiative Forcing Intercomparison Project and RSW = Reflected Shortwave radiation. Trend computed for anomalies time series (Δ).

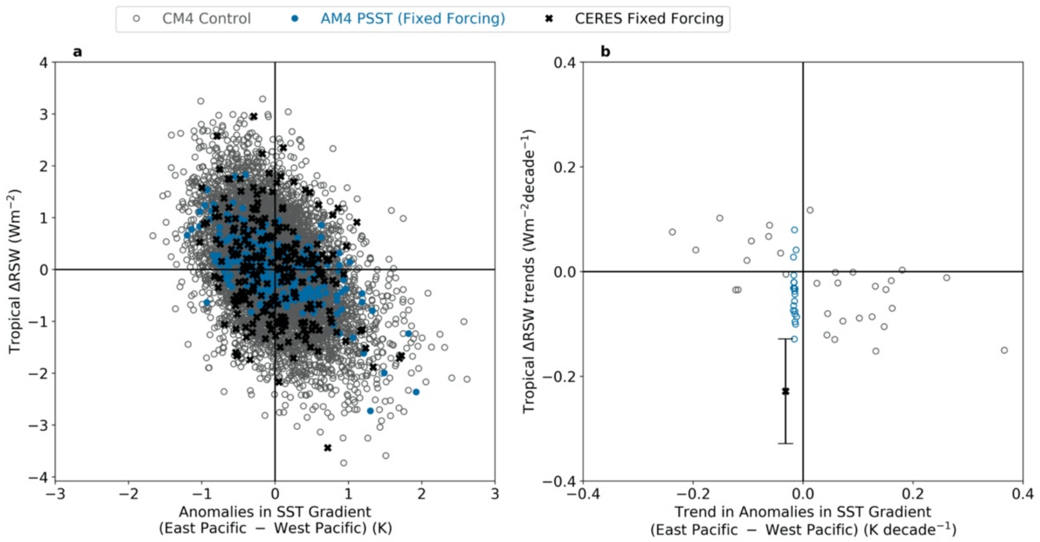

Supplementary Figure 10| Tropical pattern effect. a, Monthly mean anomalies (Δ) for CERES and CM4 and ensemble-mean monthly-means of AM4 are plotted. **b,** Same as **a** but in trends units. Monthly means in CERES, CM4, and AM4 exhibit a negative relationship between changes in the zonal SST gradient and tropical albedo. However, this is not the case for decadal trends. The 2001-2020 period shows a neutral warming pattern (slightly more West Pacific warming than East Pacific warming), yet, there is a large reduction in RSW. Trends are area-weighted (CERES error bar given by observational uncertainty). See Supplementary Table 6 for slopes and uncertainty quantification. CERES Fixed Forcing = AM4 ERF has been removed from CERES, RSW = Reflected Shortwave radiation, CERES = Clouds and the Earth's Radiant Energy System satellite observations (black), AM4 PSST = Prescribed sea surface temperatures (SSTs) and sea ice with forcing agents held fixed at 2014 levels in GFDL AM4 model (blue).

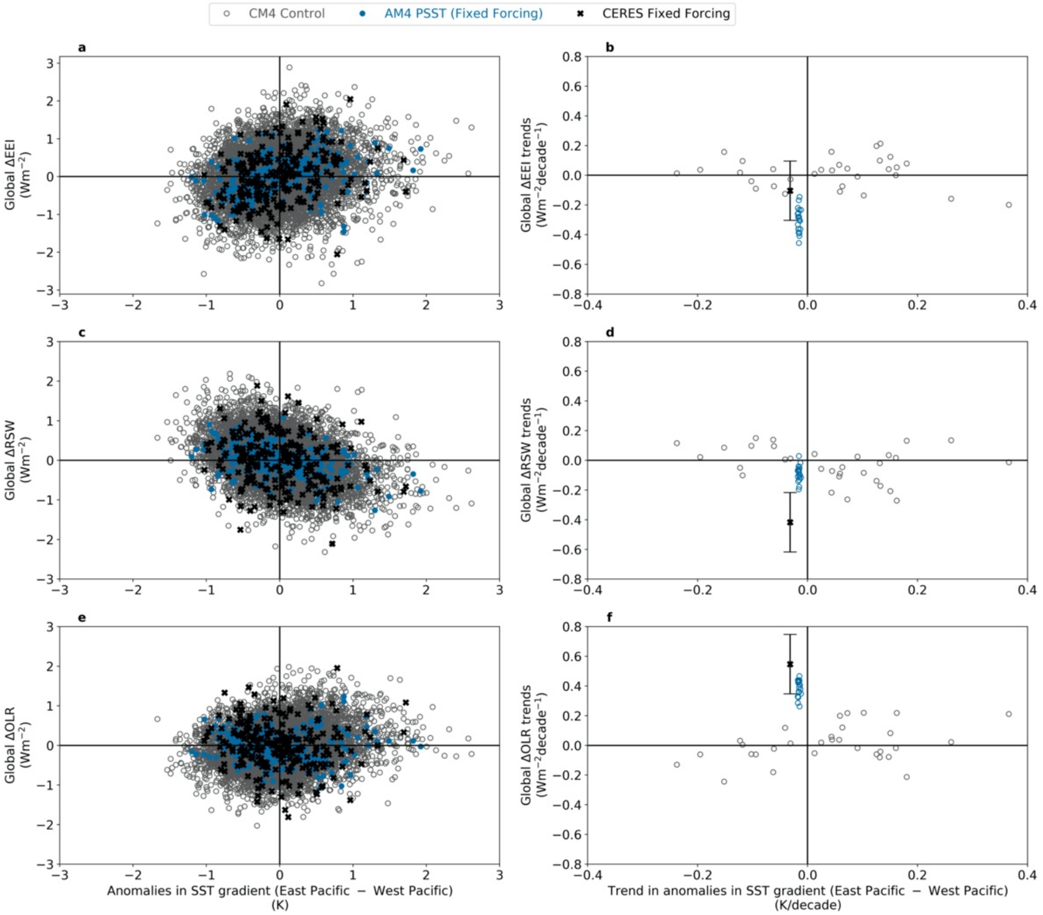

Supplementary Figure 11| Relation between global top-of-atmosphere radiation changes and East-West tropical Pacific SST gradient. a, Global EEI (Earth's Energy Imbalance). **b,** Same as **a,** but in trends units. **c-d,** Same as **a-b** but global RSW (Reflected Shortwave Radiation). **e-f,** Same as **a-b** but global OLR (Outgoing Longwave Radiation). CERES error bar in b, d, f given by observational uncertainty. CERES Fixed Forcing = AM4 ERF has been removed from CERES, RSW = Reflected Shortwave radiation, CERES = Clouds and the Earth's Radiant Energy System satellite observations (black), AM4 PSST = Prescribed sea surface temperatures (SSTs) and sea ice with forcing agents held fixed at 2014 levels in GFDL AM4 model (blue). Anomalies denoted by Δ. Trend computed for anomalies time series (Δ).

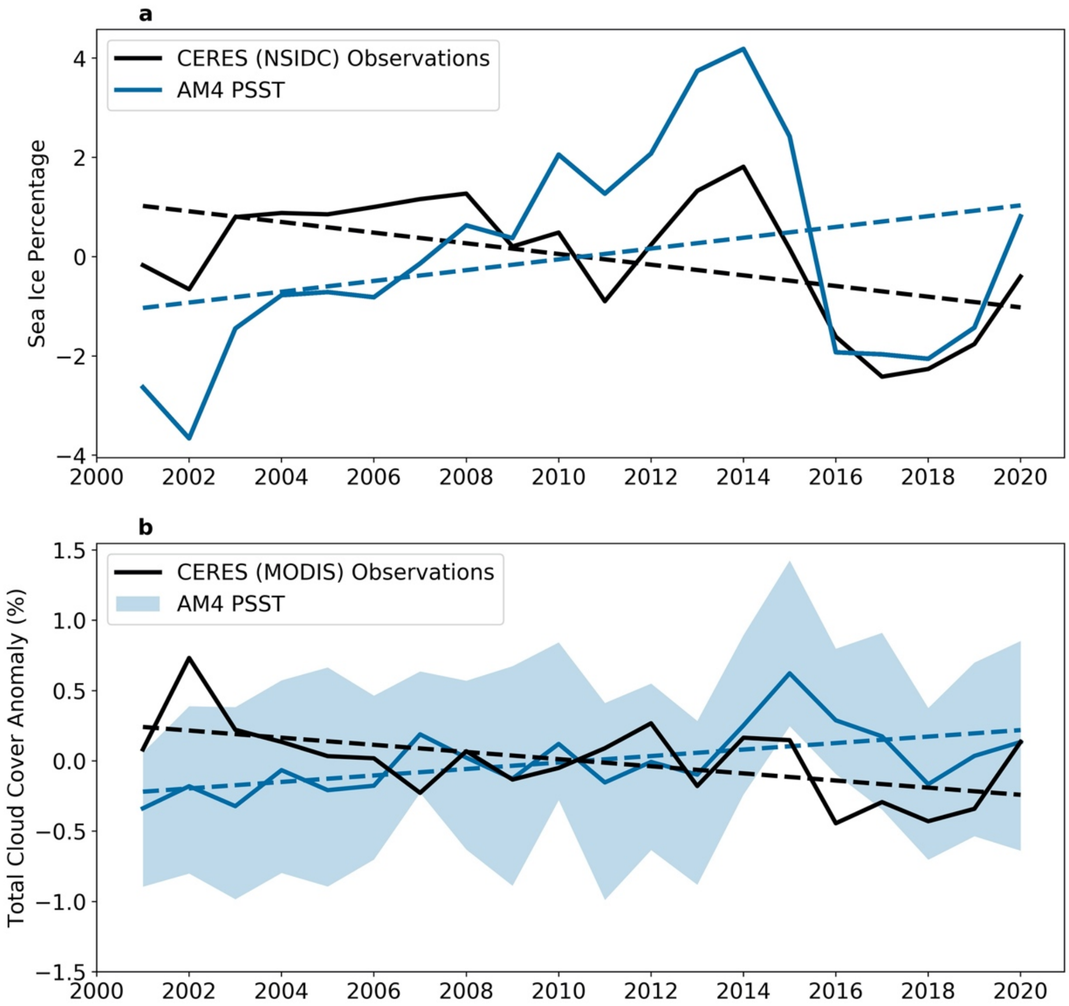

Supplementary Figure 12| Model-observation biases. a, Sea ice percentage anomaly time series over 55S-65S. **b,** Total cloud cover anomaly time series over 45S-55S. Blue shading represents full range of 20 time series realizations. Dotted lines indicate trends. Note model and observations are trending in opposite directions in **a** and **b**. CERES = Clouds and the Earth's Radiant Energy System satellite observations (black), NSIDC = National Snow and Ice Data Center, MODIS = Moderate Resolution Imaging Spectroradiometer, AM4 PSST = Prescribed sea surface temperatures (SSTs) and sea ice with forcing agents held fixed at 2014 levels in GFDL AM4 model (blue).

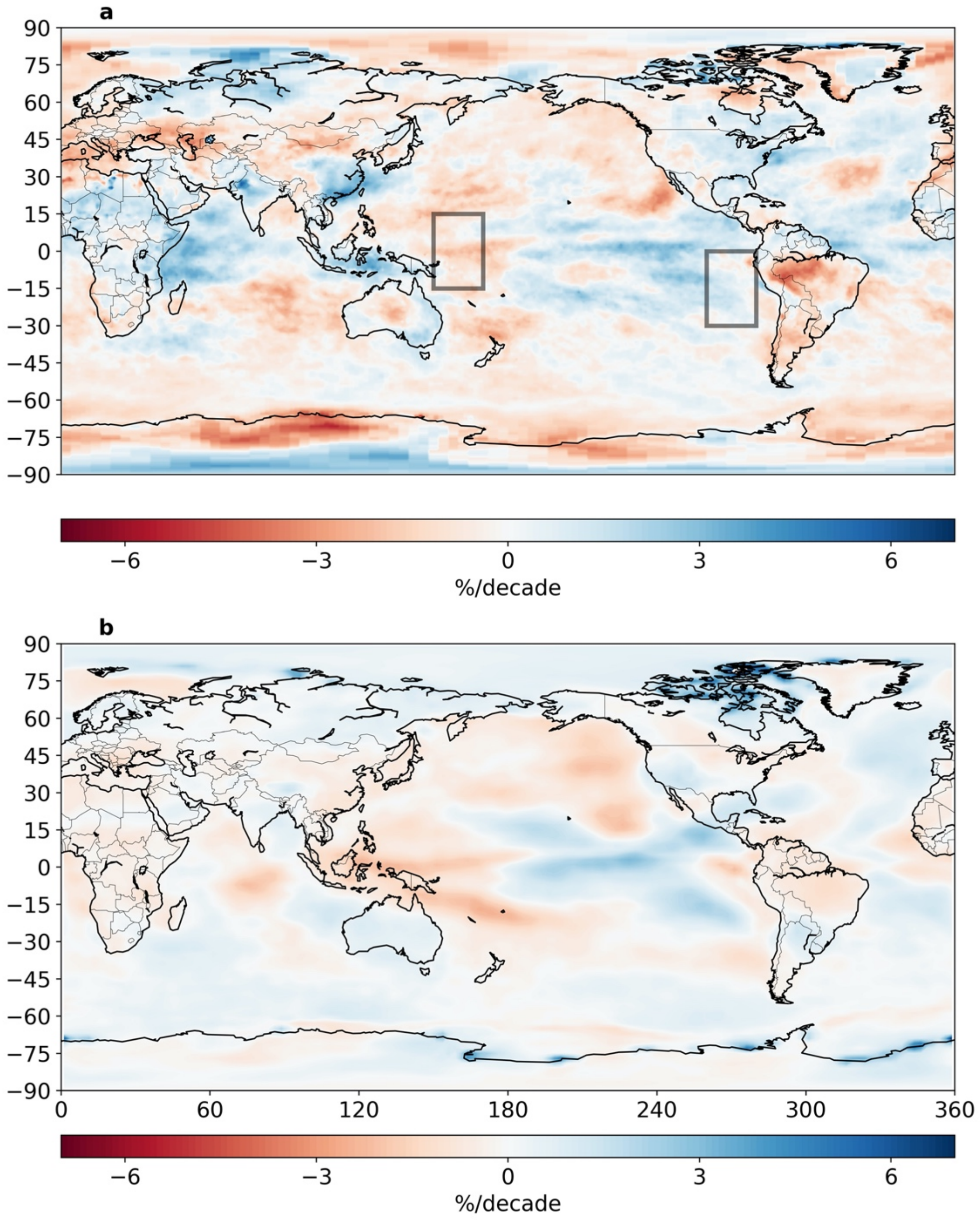

Supplementary Figure 13| Cloud fraction trends, 2001-2020. a, CERES-MODIS. **b,** AM4 PSST ensemble mean. Boxes indicate West Pacific and East Pacific areas in Pacific SST gradient of Andrews and Webb, 2018. CERES = Clouds and the Earth's Radiant Energy System satellite observations, MODIS = Moderate Resolution Imaging Spectroradiometer, AM4 PSST = Prescribed sea surface temperatures (SSTs) and sea ice with forcing agents held fixed at 2014 levels in GFDL AM4 model.

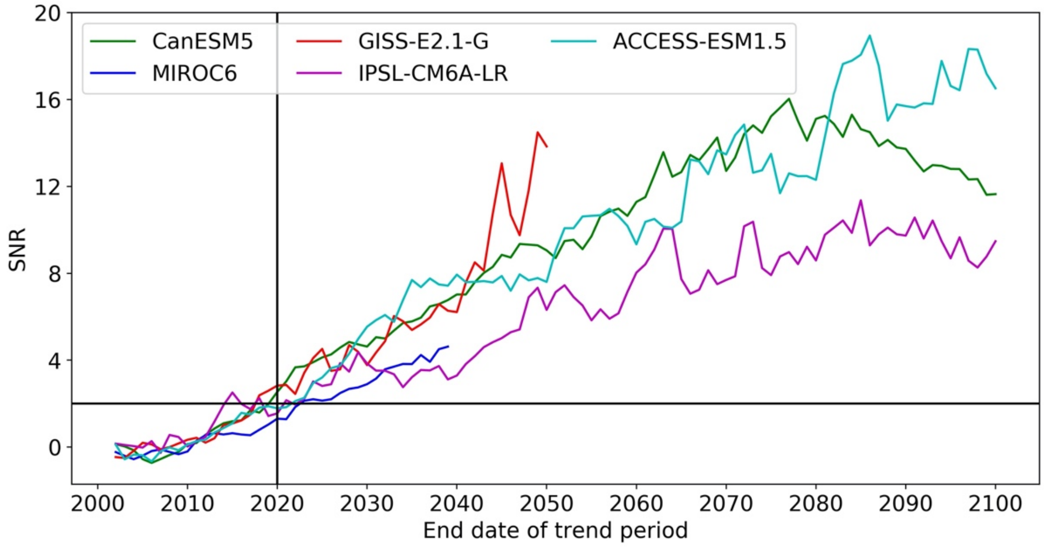

Supplementary Figure 14| Signal-to-noise ratio (SNR) for CMIP6 Historical large ensembles. $\text{SNR} = \frac{\overline{\text{Trends}}}{\sigma_{\text{Trends}}}$, where the overbar denotes the mean and σ denotes the standard deviation of the trends in each model large ensemble. Note that 3/5 of the models emerge above internal variability (SNR ≥ 2; denoted by horizontal black line) during 2001-2020. The other 2 models (MIROC6 and ACCESS-ESM1.5) emerge shortly after 2020, in 2022-2023. CanESM5 = Canadian Earth System Model version 5, MIROC6 = Model for Interdisciplinary Research on Climate version 6, IPSL-CM6A-LR = Institut Pierre-Simon Laplace-Climate Model 6-Low Resolution, ACCESS-ESM1.5 = Australian Community Climate and Earth System Simulator Earth System Model Version 1.5, GISS-E2.1-G = Goddard Institute for Space Studies (GISS) climate model.

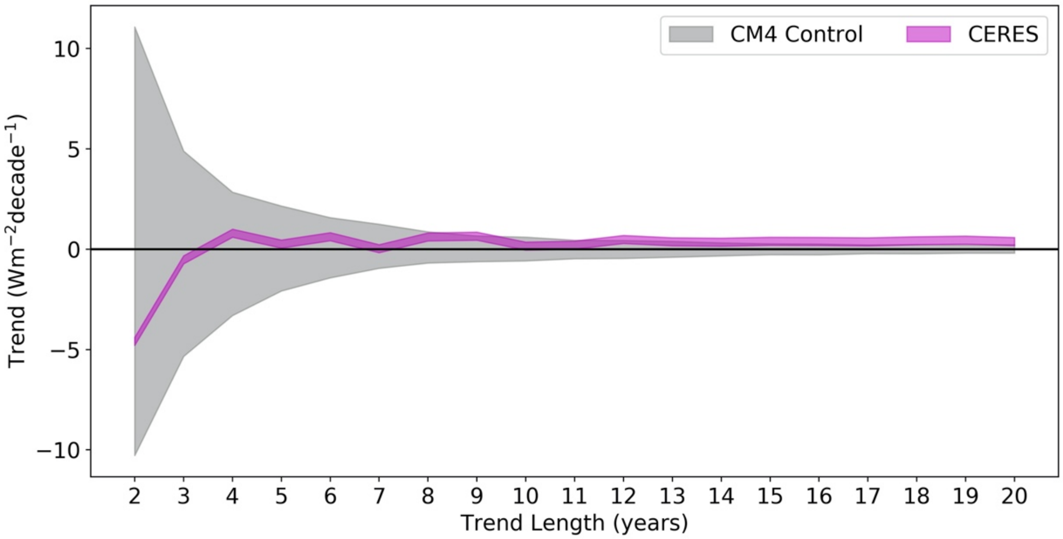

Supplementary Figure 15| Trend value as a function of trend length. Model shading represents ϵ, i.e., 2σ spread of trends. CERES shading represents observational uncertainty. CERES = Clouds and the Earth's Radiant Energy System satellite observations and CM4 = GFDL Coupled Model 4.

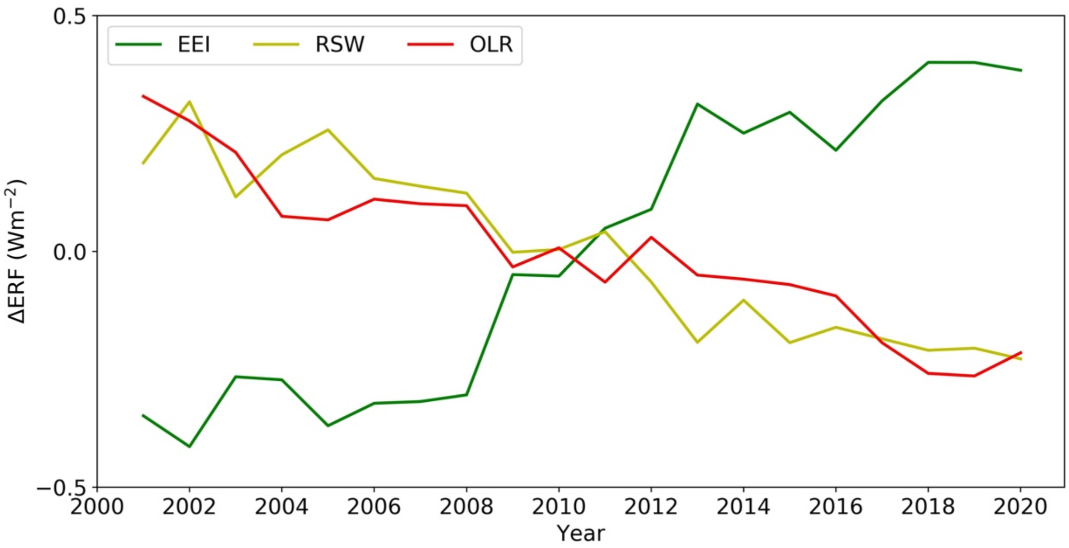

Supplementary Figure 16| Effective Radiative Forcing (ERF) time series. Calculated as the ensemble mean of the difference between the AM4 PSST+ERF and AM4 PSST ensembles. Positive for EEI indicates more energy into the system (vice-versa for RSW and OLR). AM4 PSST = Prescribed sea surface temperatures (SSTs) and sea ice with forcing agents held fixed at 2014 levels in GFDL AM4 model. AM4 PSST+ERF = GFDL AM4 with Prescribed SSTs and sea ice with forcing agents varying. EEI = Earth's Energy Imbalance, RSW = Reflected Shortwave Radiation, and OLR = Outgoing Longwave Radiation. Anomalies denoted by Δ.

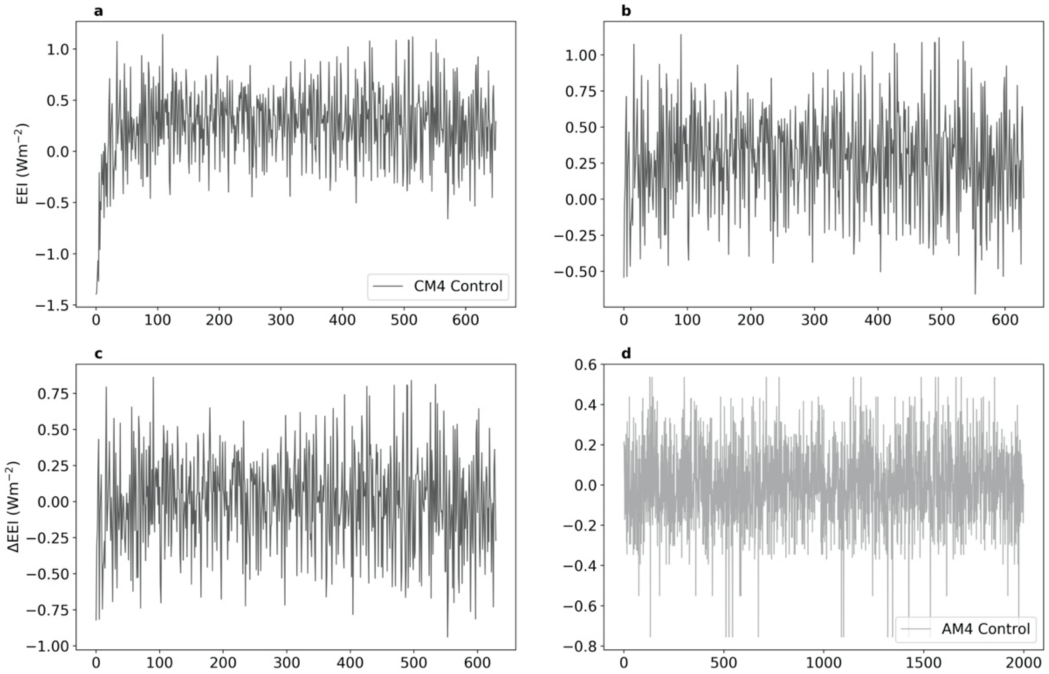

Supplementary Figure 17| a, CM4 Control simulation full 650 years. **b,** Same as **a** but excludes first 20 years and last 2 years. **c,** Same as **b** but in terms of anomalies (Δ) about time series mean of 0.28 Wm^{-2}. **d,** AM4 Control simulation full 2000 years anomaly time series. CM4 = GFDL Coupled Model 4. EEI = Earth's Energy Imbalance.

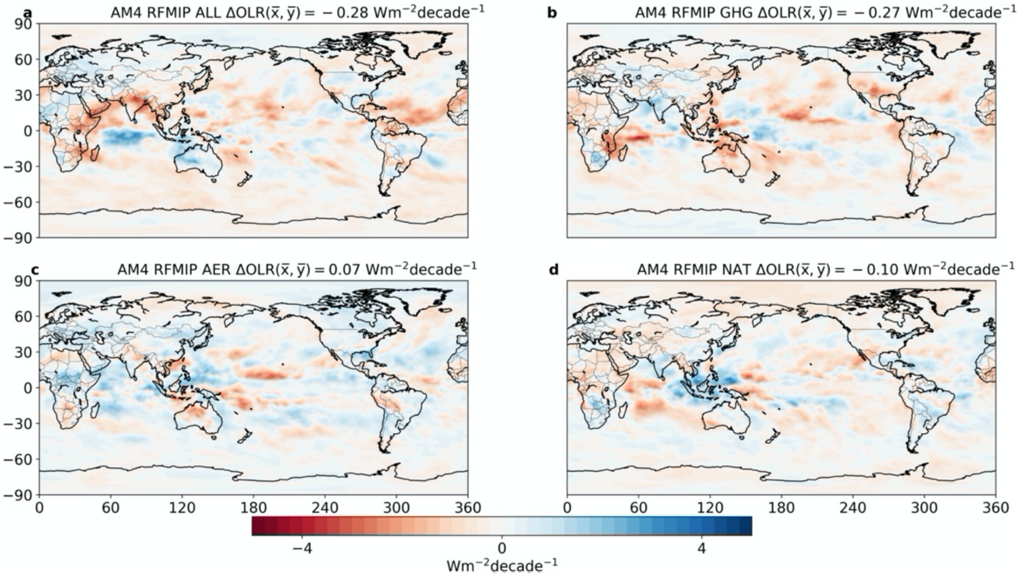

Supplementary Figure 18| Same as Supplementary Figure 9 but for OLR. Positive indicates less energy in the system.

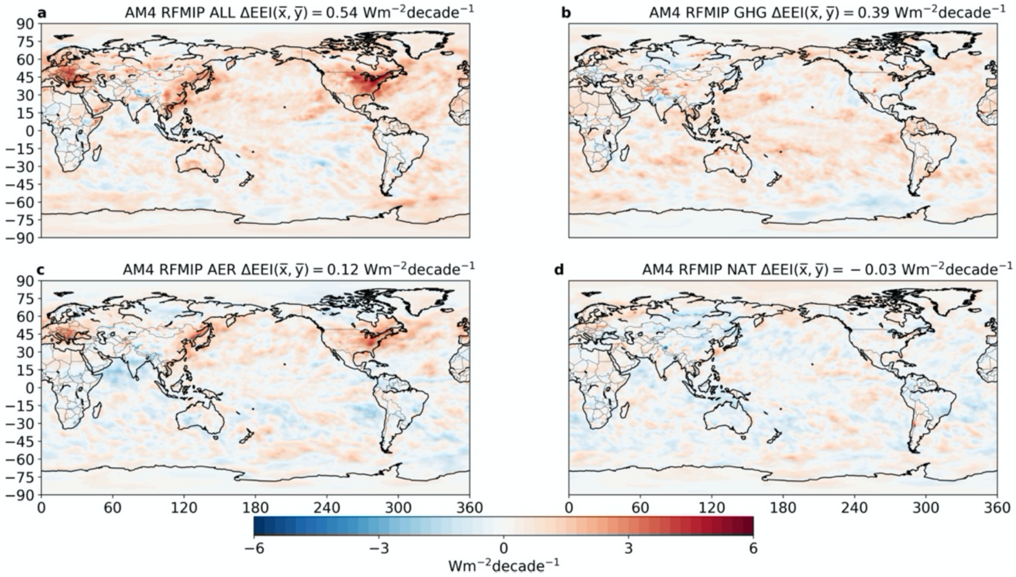

Supplementary Figure 19| Same as Supplementary Figure 9 but for EEI. Positive indicates more energy in the system.

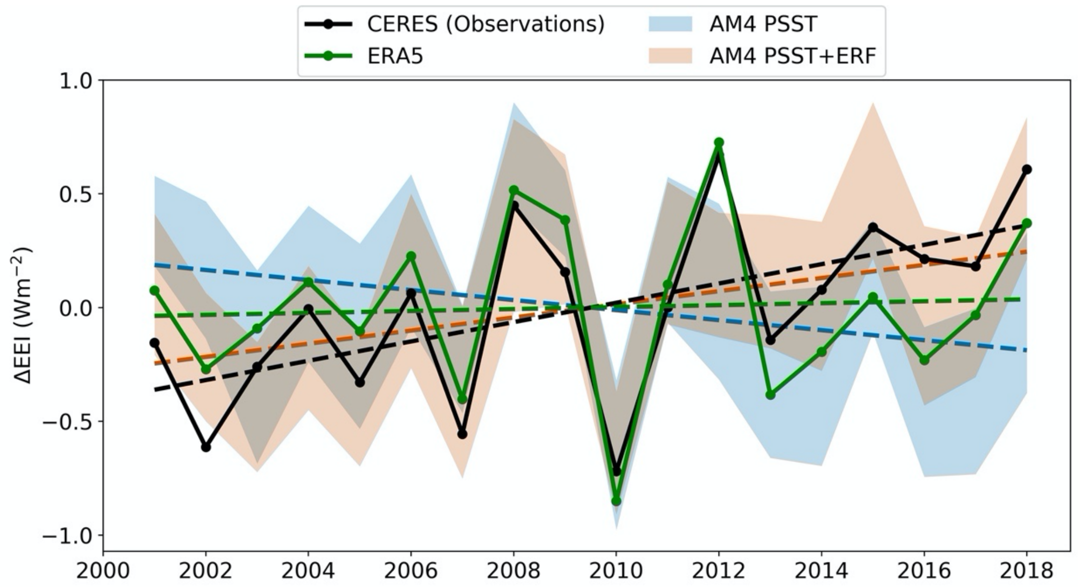

Supplementary Figure 20| ERA5 reanalysis does not reproduce CERES nor AM4 PSST+ERF trends. ERA5 = European Center for Medium-Range Weather Forecasts Reanalysis 5. Shading represents full range of time series realizations (20 realizations in AM4 PSST ensemble and 20 realizations in AM4 PSST+ERF ensemble). CERES = Clouds and the Earth's Radiant Energy System satellite observations (black), AM4 PSST = Prescribed sea surface temperatures (SSTs) and sea ice with forcing agents held fixed at 2014 levels in GFDL AM4 model (blue), AM4 PSST+ERF = same as AM4 PSST but with forcing agents varying (orange). Anomalies denoted by Δ. Trend computed for anomalies time series (Δ).

Supplementary Tables:

Supplementary Table 1: CMIP6 model piControl mean and ϵ values for trends in Earth's energy imbalance (TEEI) for 20-year periods (consecutive, non-overlapping). ϵ defined as 2σ range of trends in EEI. 'n' represents number of realizations (number of 20-year periods). Each model only uses 'r1i1p1f1' realization from CMIP6 archive. Units in $\mathrm{Wm^{-2}decade^{-1}}$. Full piControl time series used for each model apart from NOAA GFDL CM4 which ignores first 20 years due to drift (see Supplementary Figure 13). Note that $\epsilon > 0.28\ \mathrm{Wm^{-2}decade^{-1}}$ does not necessarily mean that the model has a trend $> 0.28\ \mathrm{Wm^{-2}decade^{-1}}$. Only 4 realizations out of 1,293 realizations exhibit such a large trend.

	**Model**	**Mean**	**ϵ**
1.	AS-RCEC TaiESM1 (n=25)	-0.03	$\pm$0.14
2.	AWI CM1.1-MR (n=25)	-0.03	$\pm$0.26
3.	BCC CSM2-MR (n=30)	0.01	$\pm$0.18
4.	BCC ESM1 (n=22)	-0.01	$\pm$0.15
5.	CAMS CSM1.0 (n=25)	0.01	$\pm$0.19
6.	CAS FGOALS-f3-L (n=28)	0.01	$\pm$0.16
7.	CAS FGOALS-g3 (n=33)	0.01	$\pm$0.13
8.	CCCma CanESM5 (n=50)	0.00	$\pm$0.15
9.	CCCR-IITM ESM (n=10)	-0.01	$\pm$0.10
10.	CNRM CM6.1 (n=25)	-0.01	$\pm$0.12
11.	CNRM CM6.1-HR (n=15)	0.02	$\pm$0.22
12.	CNRM ESM2.1 (n=25)	0.00	$\pm$0.14
13.	CSIRO ACCESS ESM1.5 (n=45)	0.01	$\pm$0.22
14.	CSIRO ACCESS CM2 (n=25)	0.01	$\pm$0.16
15.	E3SM1.0 (n=25)	-0.01	$\pm$0.24
16.	E3SM1.1 (n=8)	-0.03	$\pm$0.18
17.	E3SM1.1-ECA (n=8)	0.02	$\pm$0.24
18.	EC-Earth 3 (n=25)	-0.02	$\pm$0.21
19.	EC-Earth3-Veg (n=25)	0.00	$\pm$0.17
20.	INM CM4.8 (n=26)	0.01	$\pm$0.15
21.	IPSL CM6A-LR (n=60)	0.00	$\pm$0.18
22.	MIROC6 (n=40)	-0.01	$\pm$0.24
23.	MIROC E2SL (n=25)	0.00	$\pm$0.14
24.	MOHC HadGEM3-GC31-LL (n=25)	0.01	$\pm$0.15
25.	MOHC HadGEM3-GC31-MM (n=25)	0.01	$\pm$0.12
26.	MOHC UKESM1-0-LL (n=37)	0.00	$\pm$0.19
27.	MPI ESM2-HR (n=25)	-0.04	$\pm$0.22
28.	MPI ESM2-LR (n=50)	-0.01	$\pm$0.19
29.	MRI ESM2.0 (n=35)	0.03	$\pm$0.15
30.	NASA GISS E2-1-G (n=42)	-0.03	$\pm$0.17
31.	NASA GISS E2-1-G-CC (n=8)	0.01	$\pm$0.16
32.	NASA GISS E2-1-H (n=40)	0.02	$\pm$0.15

33.	NASA GISS E2-2-G (n=7)	-0.03	± 0.11
34.	NCAR CESM2 (n=49)	-0.01	± 0.25
35.	NCAR CESM2-FV2 (n=25)	0.03	± 0.17
36.	NCAR CESM2-WACCM (n=24)	0.02	± 0.28
37.	NCAR CESM2-WACCM-FV2 (n=25)	-0.02	± 0.24
38.	NCC NorCPM1 (n=25)	-0.02	± 0.18
39.	NCC NorESM1-F (n=10)	0.01	± 0.17
40.	NCC NorESM2-LM (n=25)	0.01	± 0.36
41.	NCC NorESM2-MM (n=25)	-0.02	± 0.21
42.	NOAA GFDL CM4 (n=31)	0.02	± 0.20
43.	NOAA GFDL ESM4 (n=25)	-0.02	± 0.24
44.	NUIST ESM3 (n=25)	-0.01	± 0.21
45.	SNU SAMO-UNICON (n=35)	0.00	± 0.19
46.	THU CIESM (n=25)	0.00	± 0.14
47.	UA MCM UA1.0 (n=25)	0.04	± 0.15
	Multi-model mean and 95% CI	0.00 ± 0.01	$\pm 0.18 \pm 0.01$
	CMIP6 Control-wide (n=1,293)	0.00	± 0.19

Supplementary Table 2: CMIP6 Historical models. 'n' represents the number of realizations per model. Only models with 10 or more realizations (large ensemble) are analyzed so as to obtain a realistic estimate of ϵ. Note that CanESM5 and GISS-E2.1-G contain multiple physics and forcing configurations (Methods).

	**Model**	**Mean**	ϵ
1.	CCCma CanESM5 (n=50)	0.16 ± 0.02	± 0.13
	CanESM5 'p1' (n=25)	0.17 ± 0.02	± 0.12
	CanESM5 'p2' (n=25)	0.16 ± 0.03	± 0.15
2.	MIROC6 (n=50)	0.13 ± 0.03	± 0.20
3.	IPSL CM6A-LR (n=11)	0.15 ± 0.06	± 0.20
4.	CSIRO ACCESS-ESM1.5 (n=12)	0.23 ± 0.07	± 0.26
5.	NASA GISS-E2.1-G (n=19)	0.22 ± 0.04	± 0.16
	GISS-E2.1-G 'p1f2' (n=10)	0.22 ± 0.04	± 0.12
	GISS-E2.1-G 'p3f1' (n=5)	0.16 ± 0.05	± 0.12
	GISS-E2.1-G 'p5f1' (n=4)	0.32 ± 0.06	± 0.12
	Multi-model mean and 95% CI	0.18 ± 0.04	$\pm 0.19 \pm 0.04$
	CMIP6 Historical-wide (n=142)	0.16 ± 0.02	± 0.20

Supplementary Table 3: CMIP6 RFMIP effective radiative forcing trends during 2001-2020. Each model only uses 'r1i1p1f1' realization from CMIP6 RFMIP archive apart from NOAA GFDL CM4 which we have listed the 3-member ensemble mean. Units in $\mathrm{Wm^{-2}decade^{-1}}$.

	**Model**	**Effective Radiative Forcing Trend**
1.	CCCma CanESM5	0.49
2.	IPSL CM6A-LR	0.45
3.	MOHC HadGEM3-GC31-LL	0.59
4.	MIROC6	0.36
5.	NASA GISS E2-1-G	0.58
6.	NOAA GFDL CM4	0.54
	Multi-model mean and 95% CI	0.50 ± 0.07

Supplementary Table 4: GFDL CM4 effective radiative forcing trends for clear-sky OLR and RSW during 2001-2020. Units in $\mathrm{Wm^{-2} decade^{-1}}$. Uncertainty given by 95% CI for the 3 realizations.

Effective Radiative Forcing Trend	All forcing	All forcing	GHG only	AER only	NAT only
	AM4 ERF	RFMIP	RFMIP	RFMIP	RFMIP
RSW Clear	-0.05 ± 0.01	-0.13 ± 0.01	-0.03 ± 0.03	-0.12 ± 0.03	0.08 ± 0.02
OLR Clear	-0.35 ± 0.01	-0.37 ± 0.04	-0.36 ± 0.03	0.05 ± 0.01	-0.08 ± 0.04

Supplementary Table 5| Breakdown of AM4 PSST's global RSW trend spatially and into clear-sky and cloud radiative effect components. Note the dominance of the Tropics in all-sky RSW. All-sky values are identical to Supplementary Figure 2 values. Uncertainty given by 95% CI. Units in $\mathrm{Wm^{-2}decade^{-1}}$. Tropical trends are area weighted. Positive values for RSW and SWCRE indicate less energy in the system.

Quantity	Global	Tropics	N. Ext.	S. Ext.	Poles
Clear-sky	-0.14 ± 0.01	-0.02 ± 0.01	-0.03 ± 0.01	0.00 ± 0.00	-0.09 ± 0.01
CRE	0.05 ± 0.02	-0.02 ± 0.02	-0.01 ± 0.01	0.03 ± 0.01	0.05 ± 0.01
All-sky	-0.09 ± 0.02	-0.04 ± 0.02	-0.04 ± 0.01	0.03 ± 0.01	-0.03 ± 0.01

Supplementary Table 6| Relation between monthly anomalies of top-of-atmosphere radiation vs. monthly anomalies in Andrews and Webb, 2018 East-West Pacific sea surface temperature gradient metric. Clear-sky OLR has the following regression slopes for global and tropical means respectively: CERES Fixed Forcing: -0.10 ± 0.10; 0.04 ± 0.15 and AM4 PSST (Fixed Forcing): 0.00 ± 0.12; 0.13 ± 0.03. Forcing variations (estimated via AM4 PSST+ERF-AM4 PSST) are removed from CERES. AM4 PSST uses all 20 realizations in its slope calculation, not just the ensemble mean. See Supplementary Figure 10-S11 for the ensemble mean plotted. Uncertainty given by 95% CI. Tropical all-sky and clear-sky OLR both increase with more East Pacific warming, which is contrary to the current prevailing view (that it should be negative).

Regression slope ($Wm^{-2}K^{-1}$)	N		RSW		OLR	
	Global	Tropical	Global	Tropical	Global	Tropical
CM4 Control	0.22 ± 0.03	0.30 ± 0.05	-0.40 ± 0.02	-0.82 ± 0.04	0.19 ± 0.02	0.52 ± 0.04
AM4 Fixed Forcing	0.35 ± 0.04	0.50 ± 0.06	-0.32 ± 0.03	-0.69 ± 0.04	-0.04 ± 0.03	0.17 ± 0.05
CERES Fixed Forcing	0.43 ± 0.17	0.45 ± 0.26	-0.32 ± 0.16	-0.62 ± 0.24	-0.11 ± 0.15	0.17 ± 0.24

Supplementary Table 7| Breakdown of all quantities' global trends into clear-sky and cloud radiative effect components. All-sky values are identical to Supplementary Figure 2 global values. Uncertainty given by 95% CI. Units in $\mathrm{Wm^{-2}decade^{-1}}$. Positive values for OLR, LWCRE, RSW, and SWCRE indicate less energy in the system. Positive values for Net and Net CRE indicate more energy in the system. Period considered here is January 2001-November 2020 because CERES EBAF EF4.1 "t" clear-sky fluxes are available only till November 2020. This version of clear-sky fluxes is comparable with how climate models compute clear-sky fluxes. Note that even though clear-sky RSW trend dominates the all-sky RSW trend in AM4 PSST, as a result of cloud masking in the poles, the total all-sky polar trend is diminished (Supplementary Table 5 for spatial breakdown and Supplementary Figure 2).

Quantity	CERES	AM4 PSST	AM4 PSST+ERF
Clear-sky OLR	-0.02 ± 0.21	0.38 ± 0.02	0.03 ± 0.02
LW CRE	0.30 ± 0.21	0.00 ± 0.01	0.09 ± 0.01
All-sky OLR	0.28 ± 0.22	0.39 ± 0.02	0.13 ± 0.02
Clear-sky RSW	-0.37 ± 0.21	-0.14 ± 0.01	-0.20 ± 0.01
SW CRE	-0.34 ± 0.23	0.05 ± 0.02	-0.18 ± 0.02
All-sky RSW	-0.71 ± 0.23	-0.09 ± 0.02	-0.38 ± 0.03
Clear-sky Net	0.35 ± 0.22	-0.24 ± 0.02	0.10 ± 0.02
Net CRE	0.05 ± 0.23	-0.06 ± 0.03	0.09 ± 0.03
All-sky Net	0.40 ± 0.24	-0.30 ± 0.03	0.19 ± 0.04

Supplementary Table 8| Northern Hemisphere vs Southern Hemisphere trends. Trends (all-sky) are area weighted.

Quantity	CERES	AM4 PSST	AM4 PSST+ERF
SH (OLR)	0.11 ± 0.24	0.14 ± 0.02	0.01 ± 0.02
NH (OLR)	0.17 ± 0.25	0.25 ± 0.03	0.12 ± 0.02
SH (RSW)	-0.30 ± 0.24	0.01 ± 0.03	-0.06 ± 0.02
NH (RSW)	-0.41 ± 0.25	-0.10 ± 0.02	-0.31 ± 0.02
SH (EEI)	0.17 ± 0.25	-0.14 ± 0.02	0.03 ± 0.02
NH (EEI)	0.21 ± 0.24	-0.16 ± 0.02	0.15 ± 0.02
